# Patient-related risk factors for late rectal bleeding after hypofractionated radiotherapy for localized prostate cancer: a single-center retrospective study

**DOI:** 10.1186/s13014-022-01998-4

**Published:** 2022-02-09

**Authors:** Tae Gyu Kim, Byungdo Park, Yun Gyu Song, Hyoun Wook Lee, Tae Hee Oh, Dong-Soo Ryu, Seung Chan Jeong, Daehyeon Cho, Jieun Oh, Kwang Min Kim, Jung Won Lee, Hyoun Soo Lee, Sung Min Kong, Jun Young Kim, Haeyoung Kim

**Affiliations:** 1 Department of Radiation Oncology, Samsung Changwon Hospital, Sungkyunkwan University School of Medicine, Changwon, 630-522 South Korea; 2 Department of Radiology, Samsung Changwon Hospital, Sungkyunkwan University School of Medicine, Changwon, 630-522 South Korea; 3 Department of Pathology, Samsung Changwon Hospital, Sungkyunkwan University School of Medicine, Changwon, 630-522 South Korea; 4 Department of Urology, Samsung Changwon Hospital, Sungkyunkwan University School of Medicine, Changwon, 630-522 South Korea; 5 Department of Internal Medicine, Samsung Changwon Hospital, Sungkyunkwan University School of Medicine, Changwon, 630-522 South Korea; 6 Departments of Radiation Oncology, Samsung Medical Center, Sungkyunkwan University School of Medicine, Seoul, 06351 South Korea

**Keywords:** Prostate cancer, Proctitis, Radiation dose hypofractionation, Anticoagulants, Liver cirrhosis

## Abstract

**Background:**

Several studies have reported patient-related risk factors for late rectal bleeding following conventionally fractionated radiotherapy for prostate cancer. We investigated patient-related risk factors for late rectal bleeding after hypofractionated radiotherapy.

**Methods:**

A total of 231 patients with local or locally advanced prostate cancer treated with hypofractionated radiotherapy (70 or 67.2 Gy in 28 fractions) were evaluated retrospectively. All patients received intensity-modulated radiotherapy with daily image guidance. The relationships between late rectal bleeding and risk factors like diabetes, hypertension, cirrhosis, and anticoagulant use were analyzed.

**Results:**

During a median follow-up of 23 months, the crude rates of grade ≥ 1, grade ≥ 2, and grade ≥ 3 late rectal bleeding were 23.8%, 16.9%, and 9.5%, respectively. Cirrhosis and anticoagulant use predicted an increased risk of grade ≥ 3 rectal bleeding in multivariable analyses (hazard ratio [HR] 14.37, 95% confidence interval [CI] 3.09–66.87, *P* = 0.001, and HR 2.93, 95% CI 1.14–7.55, *P* = 0.026, respectively). The non-anticoagulant group had a significantly superior 5-year freedom from grade ≥ 3 bleeding compared to the anticoagulant group in a propensity-weighted log-rank analysis (88.0% vs. 76.7%, *P* = 0.041). A receiver operating characteristic curve analysis revealed that rectal bleeding was minimized in the anticoagulant group if the equivalent dose at fractionation of 2 Gy (EQD2) V77 Gy of the rectum was < 4.5% or if the EQD2 V8.2 Gy was < 71.0%.

**Conclusions:**

Patients taking anticoagulants or those with cirrhosis had a significantly higher risk of severe late rectal bleeding than other patients after hypofractionated radiotherapy for prostate cancer in the present study. The bleeding risk could be lowered by minimizing hotspots in patients taking anticoagulants.

**Supplementary Information:**

The online version contains supplementary material available at 10.1186/s13014-022-01998-4.

## Background

External beam radiotherapy offers excellent long-term disease control in patients with localized prostate cancer and is currently accepted as one of the standard treatments [1, 2]. Recent large-scale randomized studies have reported that hypofractionated radiotherapy, which provides larger doses per fraction than conventionally fractionated radiotherapy, led to non-inferior outcomes compared to the conventional protocol in terms of biochemical or clinical failure and toxicity [3–5]. The theoretical background for hypofractionated radiotherapy is that prostate cancer has a low α/β value (the dose at which the linear and quadratic components of cell killing are equal), which means that using large doses per fraction increases the therapeutic effect [6–8]. In addition, hypofractionated radiotherapy improves patient convenience and lowers medical costs by reducing the number of fractions and enabling shorter treatment periods; thus, its clinical adoption is increasing.

Chronic proctitis, which can occur after curative radiotherapy for local or locally advanced prostate cancer, is a major problem that has a profound effect on a patient’s quality of life. Chronic rectal symptoms include rectal bleeding, loose stools, urgency, pelvic pain, and tenesmus, with intermittent bleeding being the most common. It occurs a median of 8 to 12 months after the end of radiotherapy, and the incidence rate is 2%–20% in studies of patients with pelvic malignancies who have received external beam radiotherapy or brachytherapy [9, 10].

For prostate cancer patients deemed to have a high likelihood of severe rectal complications, careful attempts must be made to reduce the rectal radiation dose, or treatment options other than radiotherapy have to be considered. Several previous studies of the patient characteristics associated with chronic radiation proctitis have reported anticoagulant use, patient age, a history of myocardial infarction or congestive heart failure, and diabetes as significant factors [11–17]. In particular, the relative risk of late rectal bleeding in patients on anticoagulants was as high as 2.5–4.8 [12–14].

However, most of the patients included in those studies received conventionally fractionated radiotherapy. Fraction size is known to be the dominant factor in determining late radiation-induced toxicity in normal organs [18]. The incidence and risk factors of late rectal bleeding after hypofractionated radiotherapy might differ from those seen after conventionally fractionated radiotherapy. Therefore, we investigated the patient-related risk factors for late rectal bleeding after hypofractionated radiotherapy for prostate cancer, focusing on anticoagulant use.

## Methods

This study considered patients who received curative radiotherapy for local or locally advanced prostate cancer from May 2013 to February 2020 at Samsung Changwon Hospital in Changwon, Korea. The inclusion criteria were no evidence of distant metastasis, ≥ 3 months of follow-up, and completion of scheduled radiotherapy. A total of 231 patients who met those criteria were included and analyzed. The patient medical records were reviewed retrospectively with institutional review board (IRB) approval (IRB no. SCMC 2020–11-003–001).

After deciding to receive radiotherapy, all patients proceeded to the simulation process. A rectal enema was performed about 2 h before the simulation and each treatment. In addition, to keep the bladder volume constant, patients were instructed to drink about 300 mL of water after voiding about 30 min before the simulation and each treatment. To reduce radiation-induced rectal toxicity and infra-fractional prostate motion, a rectal balloon made in house was inserted during the simulation and each treatment [19, 20]. Simulation computed tomography images were taken at a 2.5 mm slice thickness with a contrast dye injection (IOBRIX®inj.350; Taejoon Pharm Co., Ltd., Seoul, Korea). Patients were positioned in a supine position using an immobilization device in which the rectal balloon was fixed. Then, each patient underwent a magnetic resonance image test with a slice thickness of 2.5 mm on T2 axial image sets using the same preparation and posture used during the computed tomography simulation. Digital images from the computed tomography and magnetic resonance imaging scans were transferred to an Eclipse treatment planning system (version 11.0, Varian Medical Systems, Palo Alto, CA, USA) and fused for target delineation. The clinical target volume was defined as the entire prostate with or without seminal vesicles in definitive radiotherapy and as the prostate bed in adjuvant or salvage radiotherapy shown on the T2 axial image. Seminal vesicles were included in the target volume when there was pathologic or radiologic evidence of their involvement. The prostate planning target volume (PTV) was extended from the clinical target volume using an 8 mm margin in the superior–inferior direction and a 3 mm margin in the other directions while taking prostate movement into account. Regional lymphatic areas were also included in the target volume in patients with clinical evidence of lymph node metastasis or with a high probability of lymph node metastasis derived from the Roach formula [21]. The rectal wall was contoured from a level 10 mm below the lower PTV edge to a level 10 mm above the upper PTV edge. The protocol stipulates that ≥ 95% of the PTV receive the prescribed dose. A maximum dose of 107% was allowed to < 2% of the PTV. The treatment schedule consisted of 70 Gy in 28 fractions (2.5 Gy/fraction) for definitive radiotherapy and 67.2 Gy (2.4 Gy/fraction) for adjuvant or salvage radiotherapy. When regional lymphatic areas were included in the target volume, the simultaneous integrated boost technique was used with a dose of 50.4 Gy (1.8 Gy/fraction) scheduled for the nodal PTV.

Dose constraints for plan optimization were that ≤ 25% and ≤ 50% of the bladder should receive > 60 and > 35 Gy, respectively, and ≤ 7%, ≤ 20%, ≤ 50%, and ≤ 90% of the rectal wall should receive > 70, > 50, > 25, and > 12 Gy, respectively, with a maximum dose of 74 Gy. Both static intensity-modulated radiation therapy and volumetric-modulated arc therapy plans were generated for each patient, and the plan with superior dosimetric quality in terms of normal organ dose, target homogeneity, and conformity was selected for treatment. Image-guided radiation therapy was performed using pretreatment cone-beam computed tomography imaging to reduce geometric uncertainty regarding the patient setup and rectal balloon position. The rectal balloon was always inserted at the same depth in the same patient and used as a surrogate for prostate location in the pretreatment verification. A fiducial maker was not implanted for prostate localization.

Patients were followed up every 3–6 months for the first 5 years and then yearly thereafter. All patients were asked about rectal symptoms at each follow-up visit. In patients with rectal bleeding, endoscopy was recommended, and, depending on the severity, observation, steroid suppositories, sucralfate enemas, or cauterizations were performed. A careful retrospective inspection of the medical records was performed to find late rectal bleeding that appeared ≥ 90 days after radiotherapy. Toxicities were graded with reference to the European Organization for Research and Treatment of Cancer/Radiation Therapy Oncology Group (RTOG) morbidity scores for the rectum. Rectal bleeding was graded as follows: grade 1, minor or infrequent rectal bleeding improving without any intervention; grade 2, intermittent bleeding that required medication; grade 3, bleeding that required an invasive intervention, which typically included cauterization; grade 4, bleeding that required a transfusion or surgery; grade 5, bleeding associated with death. Endoscopists at our hospital were active in performing endoscopy to confirm radiation proctitis, and, thus, all patients with grade ≥ 2 toxicity and most patients with grade 1 toxicity were assessed via sigmoidoscopy or colonoscopy.

A biostatistician performed the statistical analyses. All variables were tested in binary fashion, with continuous variables dichotomized by their median values, including the generalized equivalent uniformed dose (gEUD) as a rectal dose parameter. The gEUD is a single organ–specific parameter to account for the biological response according to the delivered dose distribution in that organ. In this study, gEUDs were calculated using DVHmetrics of the R statistical computing software (https://www.r-project.org/; R Foundation for Statistical Computing, Vienna, Austria).

The gEUD is defined as follows:

$$\mathrm{gEUD }={(\sum_{i}{v}_{i}{D}_{i}^{a})}^{1/a}$$

where v_i_ is the fractional organ volume receiving a dose D_i_ and $a$ is a parameter that describes the volume effect [22]. The $a$ and $\mathrm{\alpha }/\upbeta$ ratios were assumed to be 8 and 3 for the rectum, respectively [23]. Prognostic factors for rectal bleeding were evaluated using logistic regression analyses. The characteristics of the non-anticoagulant and anticoagulant groups were compared using independent Pearson's chi-squared testing. To reduce the effects of selection bias between the two groups, significant differences in patient characteristics were adjusted using an inverse probability of treatment weighting (IPTW) and propensity scoring. Kaplan–Meier curves for freedom from rectal bleeding were generated, and comparisons were made using the log-rank test. A receiver operating characteristic (ROC) curve analysis was used to test the association between rectal bleeding and the rectal wall dose. The equivalent dose in 2.0 Gy fractions (EQD2) of the 70, 50, 25, and 12 Gy for late rectal bleeding were 77, 47.9, 19.5, and 8.2 Gy, respectively, and these values were used for ROC curve analysis. Dosimetric parameters with an area under the ROC curve (AUC) ≥ 0.6, lower 95% confidence interval value ≥ 0.5, and both sensitivity and specificity > 60% were considered reasonable predictors [24]. All statistical analyses were performed using the STATA version 15.1 software program (Stata Corporation, College Station, TX, USA) and R software program (Additional file 1: Table S1). A two-sided *P* value < 0.05 was considered to be statistically significant.

## Results

The patient characteristics are shown in Table 1. Of the 231 patients, 167 had high- or very high–risk prostate cancer (72.3%) when classified using the National Comprehensive Cancer Network risk categories. There were 47, 117, and 8 patients (20.4%, 50.7%, and 3.5%) with diabetes, hypertension, and cirrhosis, respectively. Of the 80 patients (34.6%) on anticoagulants, 72 started using them before radiotherapy and 8 started using them after radiotherapy. Forty-one patients with major cardiovascular risks, such as hypertension, diabetes, and hyperlipidemia, used anticoagulants for the primary prevention of cardiovascular disease. Anticoagulants for secondary prevention were used in 22 patients with coronary artery disease, 8 with atrial fibrillation, 8 with ischemic stroke, and 1 with heart failure. Aspirin, clopidogrel, warfarin, and other anticoagulants were used in 48, 22, 4, and 12 patients, respectively, and six patients used multiple anticoagulants. Definitive radiotherapy was performed in 146 patients (69.7%), and the others received adjuvant or salvage radiotherapy. All patients underwent intensity-modulated radiotherapy, which was volumetric-modulated arc therapy in 68.8% of cases.

**Table 1:** Patient characteristics

Variables	Number (%)
Age (years)	
Median	74
Range	52–90
Risk category	
Very low–low	16 (6.93)
Intermediate	48 (20.78)
High–very high	167 (72.29)
Gleason score	
2–6	33 (14.29)
7	105 (59.74)
8–10	93 (40.26)
Initial PSA concentration (μg/L)	
Median	12.1
Range	2.1–507.5
T stage	
T1	8 (3.46)
T2	73 (31.60)
T3	127 (54.98)
T4	23 (9.96)
Diabetes	
No	187 (79.65)
Yes	47 (20.35)
Hypertension	
No	114 (49.35)
Yes	117 (50.65)
Cirrhosis	
No	223 (96.5)
Yes	8 (3.5)
Anticoagulation use	
No	151 (65.4)
Yes	80 (34.6)
Whole-pelvic radiotherapy	
No	161 (69.70)
Yes	70 (30.30)
Androgen-deprivation therapy	
No	53 (22.94)
Yes	178 (77.06)
Previous surgery	
No (70 Gy)	146 (63.20)
Yes (67.2 Gy)	85 (36.80)
IMRT technique	
Static	72 (31.17)
Arc	159 (68.83)
gEUD (Gy)	
Median	58.3
Range	51.1–67.2

gEUD, generalized equivalent uniformed dose; IMRT, intensity-modulated radiation therapy; PSA, prostate-specific antigen

The median follow-up period was 23 months (range, 3–86 months). The median time to rectal bleeding was 12 months (range: 3–46 months). Grade ≥ 1, grade ≥ 2, and grade ≥ 3 rectal bleeding occurred in 61, 39, and 22 patients, respectively. Endoscopy confirmed that 6 of the 22 patients with grade 1 rectal bleeding had hemorrhoids, not radiation proctitis. All patients with grade ≥ 2 rectal bleeding were confirmed to have radiation proctitis. Therefore, grade ≥ 1, grade ≥ 2, and grade ≥ 3 rectal bleeding related to radiation proctitis occurred in 55, 39, and 22 patients (crude rates: 23.8%, 16.9%, and 9.5%), respectively. In both univariable and multivariable analyses, ≥ T3 disease, hypertension, cirrhosis, and gEUD predicted a greater risk of grade ≥ 2 rectal bleeding (hazard ratio [HR] 3.32, 95% confidence interval [CI] 1.28–8.65, *P* = 0.014; HR 2.98, 95% CI 1.32–6.69, *P* = 0.008; HR 22.37, 95% CI 3.65–36.99, *P* = 0.001; and HR 2.42, 95% CI 1.09–5.40, *P* = 0.031, respectively) (Table 2). Cirrhosis and anticoagulant use were associated with a greater risk of grade ≥ 3 rectal bleeding in both univariable and multivariable analyses (HR 14.37, 95% CI 3.09–66.87, *P* = 0.001; HR 2.93, 95% CI 1.14–7.55, *P* = 0.026, respectively).

**Table 2:** Univariable and multivariable analyses for grade ≥ 2 and grade ≥ 3 late rectal bleeding

Variables	Grade ≥ 2 rectal bleeding				Grade ≥ 3 rectal bleeding			
	Univariable		Multivariable		Univariable		Multivariable	
	HR (95% CI)	*P* value	HR (95% CI)	*P* value	HR (95% CI)	*P* value	HR (95% CI)	*P* value
Age (years)								
< 74	[Reference]				[Reference]			
≥ 74	1.17 (0.5–2.33)	0.664			1.30 (0.53–3.17)	0.564		
Risk category								
Very low–low	[Reference]				[Reference]			
Intermediate	0.81 (0.14–4.67)	0.817			0.30 (0.04–2.36)	0.255		
High–very high	1.66 (0.36–7.67)	0.517			0.85 (0.18–4.03)	0.833		
Gleason score								
2–6	[Reference]				[Reference]			
7	1.40 (0.44–4.50)	0.572			0.82 (0.21–3.31)	0.786		
8–10	1.74 (0.54–5.58)	0.351			1.34 (0.35–5.14)	0.668		
Initial PSA concentration (μg/L)								
< 10	[Reference]				[Reference]			
10–20	1.52 (0.64–3.64)	0.345			1.43 (0.49–4.15)	0.512		
≥ 20	1.96 (0.83–4.59)	0.124			1.31 (0.44–3.94)	0.437		
T stage								
T1–2	[Reference]		[Reference]		[Reference]			
≥ T3	2.38 (1.04–5.45)	0.041	3.32 (1.28–8.65)	0.014	1.94 (0.69–5.48)	0.209		
Diabetes								
No	[Reference]				[Reference]			
Yes	1.22 (0.53–2.77)	0.643			0.59 (0.17–2.09)	0.416		
Hypertension								
No	[Reference]		[Reference]		[Reference]			
Yes	2.22 (1.08–4.58)	0.031	2.98 (1.32–6.69)	0.008	1.19 (0.49–2.87)	0.701		
Cirrhosis								
No	[Reference]		[Reference]		[Reference]		[Reference]	
Yes	17.27 (3.34–89.26)	0.001	22.37 (3.65–36.99)	0.001	11.39 (2.63–49.40)	0.001	14.37 (3.09–66.87)	0.001
Whole-pelvic radiotherapy								
No	[Reference]				[Reference]			
Yes	1.56 (0.76–3.19)	0.226			1.35 (0.54–3.39)	0.517		
Androgen-deprivation therapy								
Yes	[Reference]				[Reference]			
No	1.40 (0.65–3.05)	0.393			1.29 (0.48–3.49)	0.612		
Radiation dose								
67.2 Gy	[Reference]				[Reference]			
70 Gy	1.6 (0.86–4.04)	0.117			1.28 (0.50–3.27)	0.611		
IMRT technique								
Static	[Reference]				[Reference]			
Arc	1.02 (0.49–2.16)	0.953			0.97 (0.38–2.49)	0.945		
Anticoagulation use								
No	[Reference]				[Reference]		[Reference]	
Yes	1.22 (0.60–2.49)	0.582			2.49 (1.02–6.05)	0.044	2.93 (1.14–7.55)	0.026
gEUD (Gy)								
< 58.3	[Reference]		[Reference]		[Reference]			
≥ 58.3	3.01 (1.42–6.39)	0.004	2.42 (1.09–5.40)	0.031	1.49 (0.61–3.63)	0.384		

CI, confidence interval; gEUD, generalized equivalent uniformed dose; HR, hazard ratio; IMRT, intensity-modulated radiation therapy; PSA, prostate-specific antigen

**Fig. 1:**
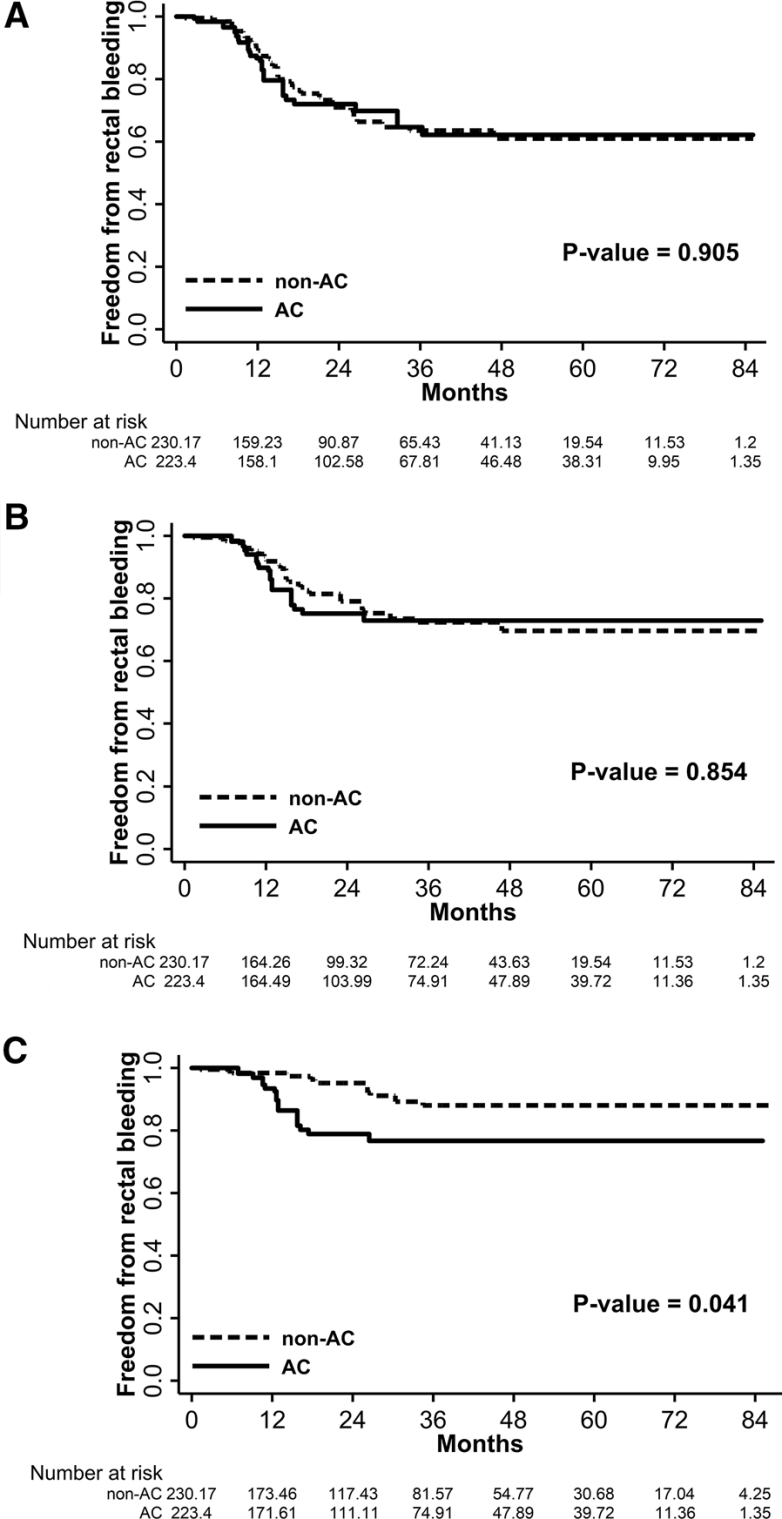
Freedom from rectal bleeding in the non-anticoagulant and anticoagulant groups as found in a propensity-weighted log-rank analysis. Results are stratified as grade ≥ 1 rectal bleeding (**a**), grade ≥ 2 rectal bleeding (**b**), and grade ≥ 3 rectal bleeding (**c**)

Because severe rectal bleeding (grade ≥ 3) could significantly affect a patient’s quality of life, further analyses were conducted on anticoagulant use. For cirrhosis, further analysis was not performed due to an insufficient number of patients. To verify the relationship between anticoagulant use and rectal bleeding, patients were divided into two groups by anticoagulant use (Table 3, Additional file 1: Table S2). Both groups had similar disease risk categories and treatment characteristics; however, patients in the anticoagulant group were older than those in the non-anticoagulant group (age ≥ 74 years: 62.5% vs. 48.3%, *P* = 0.004), and diabetes mellitus and hypertension were more common in the anticoagulant group (32.5% vs. 13.9%, *P* = 0.001; 67.5% vs. 41.7%, *P* < 0.001, respectively). Of the 151 patients in non-anticoagulant group, 24 and 10 patients developed grade ≥ 2 and grade ≥ 3 rectal bleeding (crude rates: 15.9% and 6.6%), respectively. Of the 80 patients in the anticoagulant group, grade ≥ 2 and grade ≥ 3 rectal bleeding occurred in 15 and 12 patients (crude rates: 18.8% and 15.0%), respectively. Patients on anticoagulants had a significantly higher risk of grade ≥ 3 rectal bleeding than those who did not (HR 2.49, 95% CI 1.02–6.05, *P* = 0.044). The difference in the risk of grade ≥ 3 rectal bleeding remained significant even after compensating for the main effect of all variables on anticoagulant use using IPTW methods (HR 2.94, 95% CI 1.08–7.95, *P* = 0.034). Freedom from rectal bleedings were compared between the two groups using a propensity-weighted log-rank analysis (Fig. 1). There was no significant difference in the actuarial rates of 5-year freedom from grade ≥ 1 and grade ≥ 2 bleeding between the non-anticoagulant and anticoagulant groups (61.0% vs. 62.2%, *P* = 0.905; 69.6% vs. 72.9%, *P* = 0.854, respectively). However, the non-anticoagulant group had significantly better 5-year freedom from grade ≥ 3 bleeding than the anticoagulant group (88.0% vs. 76.7%; *P* = 0.041).

**Table 3:** Comparisons between the anticoagulant and non-anticoagulant groups

Variables	Non-AC (%)	AC (%)	Before IPTW	After IPTW
			*P* value	*P* value
Age (years)			0.004	0.891
< 74	78 (51.7)	30 (37.5)		
≥ 74	73 (48.3)	50 (62.5)		
Risk category			0.106	0.947
Very low–low	9 (6.0)	7 (8.8)		
Intermediate	26 (17.2)	22 (27.5)		
High–very high	116 (76.8)	51 (63.7)		
Gleason score			0.211	0.987
2–6	18 (11.9)	15 (18.8)		
7	67 (44.4)	38 (47.5)		
8–10	66 (43.7)	27 (33.8)		
Initial PSA concentration (μg/L)			0.138	0.802
< 10	51 (33.8)	37 (46.2)		
10–20	50 (33.1)	24 (30.0)		
≥ 20	50 (33.1)	19 (23.8)		
T stage			0.085	0.613
T1–2	47 (31.1)	34 (42.5)		
≥ T3	104 (68.9)	46 (57.5)		
Diabetes			0.001	0.883
No	130 (86.1)	54 (67.5)		
Yes	21 (13.9)	26 (32.5)		
Hypertension			< 0.001	0.782
No	88 (58.3)	26 (32.5)		
Yes	63 (41.7)	54 (67.5)		
Cirrhosis			0.560	0.698
No	145 (96.0)	78 (97.5)		
Yes	6 (3.97)	2 (2.50)		
Whole-pelvic radiotherapy			0.202	0.575
No	101 (66.9)	60 (75.0)		
Yes	50 (33.1)	20 (25.0)		
Androgen-deprivation therapy			0.231	0.973
No	31 (20.5)	22 (27.5)		
Yes	120 (79.5)	58 (72.5)		
Radiation dose			0.462	0.788
67.2 Gy	53 (35.1)	32 (40.0)		
70 Gy	98 (64.9)	48 (60.0)		
IMRT technique			0.360	0.893
Static	44 (29.1)	28 (35.0)		
Arc	107 (70.9)	52 (65.0)		

AC, anticoagulant; IMRT, intensity-modulated radiation therapy; IPTW, inverse probability of treatment weighting; PSA, prostate-specific antigen

**Fig. 2:**
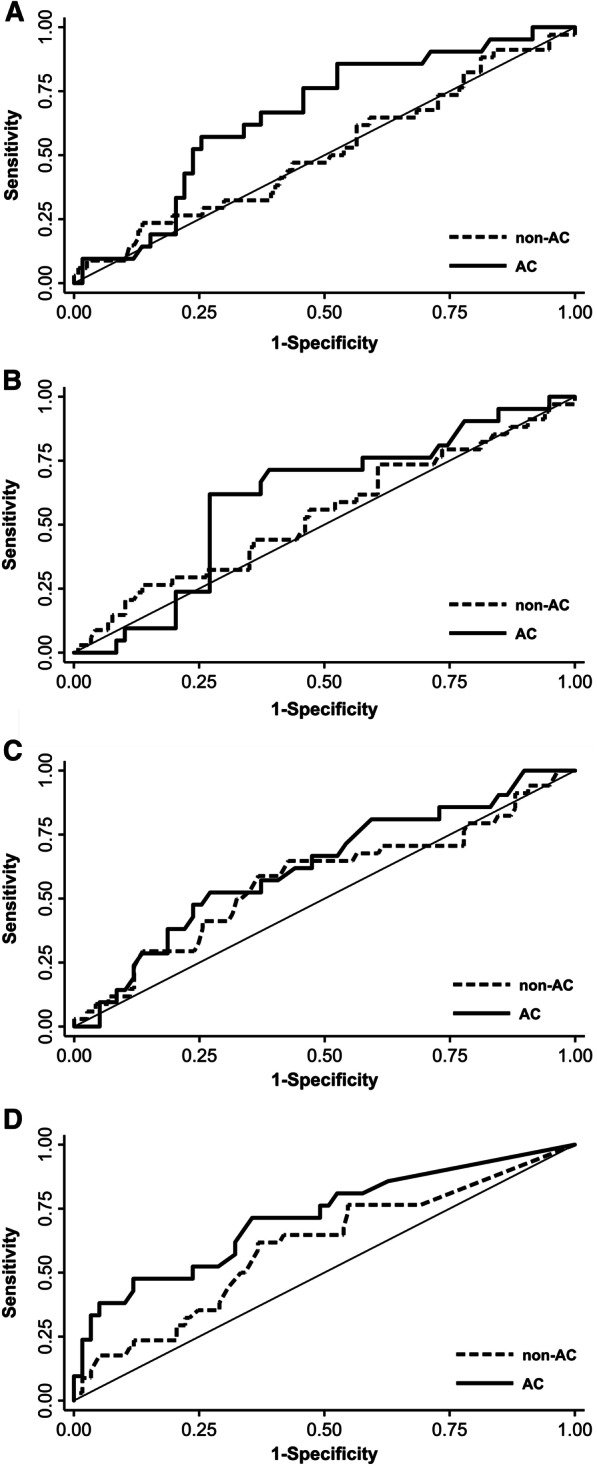
Receiver operating characteristic curve of a rectal equivalent dose at fractionation of 2 Gy V8.2 Gy (**a**), V19.5 Gy (**b**), V47.9 Gy (**c**), and V77 Gy (**d**) for predicting late rectal bleeding

We conducted a ROC curve analysis of the dosimetric parameters of the rectal wall volume in the non-anticoagulant and anticoagulant groups, respectively (Table 4). In the non-anticoagulant group, EQD2 V77 Gy had the highest AUC value (0.601) among the parameters, but the lowest confidence interval was < 0.5. Other parameters were not significant in predicting rectal bleeding in the non-anticoagulant group. All dosimetric parameters had higher AUC values in the anticoagulant group than in the non-anticoagulant group (Fig. 2). EQD2 V77 Gy (AUC = 0.715, *P* = 0.002) and EQD2 V8.2 Gy (AUC = 0.657, *P* = 0.017) significantly predicted rectal bleeding in the anticoagulant group. The AUC values of EQD2 V47.9 Gy and EQD2 V8.2 Gy were not considered reasonable predictors because their lower confidence interval value was < 0.5. The cut-off points were EQD2 V77 Gy = 4.5% (Youden’s index 0.358, sensitivity 71.4%, and specificity 64.4%) and EQD2 V8.2 Gy = 71.0% (Youden’s index 0.332, sensitivity 85.7%, and specificity 47.5%). When considering the AUC value, sensitivity, and specificity together, EQD2 V77 Gy was the most predictive parameter for rectal bleeding in the anticoagulant group.

**Table 4:** Receiver operating characteristic curve analysis of dosimetric parameters of rectal wall volume for late rectal bleeding in the anticoagulant and non-anticoagulant groups

Rectal dose (EQD2)	Non-AC			AC		
	Cutoff point	AUC (95% CI)	*P* value	Cutoff point	AUC (95% CI)	*P* value
V77 Gy	5.3%	0.601 (0.490–0.712)	0.035	4.5%	0.715 (0.579–0.850)	0.002
V47.9 Gy	18.8%	0.573 (0.455–0.690)	0.099	19.6%	0.623 (0.482–0.764)	0.048
V19.5 Gy	36.8%	0.539 (0.424–0.653)	0.247	41.9%	0.603 (0.464–0.742)	0.082
V8.2 Gy	87.4%	0.512 (0.398–0.625)	0.419	71.0%	0.657 (0.525–0.788)	0.017

AC, anticoagulant; AUC, area under the receiver operating characteristic curve; CI, confidence interval; EQD2; equivalent dose at fractionation of 2 Gy

## Discussion

In this study, the crude rates of grade ≥ 1, grade ≥ 2, and grade ≥ 3 late rectal bleeding after hypofractionated radiotherapy for localized prostate cancer were 23.8%, 16.9%, and 9.5%, respectively. Hypertension and cirrhosis predicted an increased risk of grade ≥ 2 rectal bleeding. The use of an anticoagulant and the presence of cirrhosis predicted an increase in the risk of grade ≥ 3 rectal bleeding. In particular, the crude rate of grade ≥ 3 rectal bleeding in patients on anticoagulants was 15.0%, which was significantly higher than the 6.6% rate in patients not taking anticoagulants.

Many dosimetric studies have been conducted on the risk factors for proctitis after external beam radiotherapy for prostate cancer. Early studies focusing on dose escalation showed that doses exceeding 70–74 Gy with conventional fractionation were associated with a high rate of late gastrointestinal toxicity [25, 26]. It is not yet clear whether hypofractionated radiotherapy is associated with an increase in late gastrointestinal toxicity. Franco et al. performed a meta-analysis to compare radiation toxicity in high-risk prostate cancer patients who underwent conventional fractionation (EQD2 55.1–82 Gy) and hypofractionated radiotherapy (EQD2 45–108.6 Gy) [27]. Patients who received hypofractionated radiotherapy had about 28% more late genitourinary toxicity than those who received conventionally fractionated treatment. However, the two fractionation groups did not differ in acute gastrointestinal, acute genitourinary, or late gastrointestinal toxicity. In patients with low-/intermediate-risk prostate cancer, the hypofractionation technique was associated with favorable late grade 2 gastrointestinal toxicity only with 3-dimensional conformal radiation therapy (3DCRT) (9% in the 3DCRT-Hypo group [EQD2 61.9–119.4 Gy] vs. 18% in the 3DCRT-CV group [EQD2 66–108 Gy], *P* < 0.0001) [28]. In patients who underwent intensity-modulated radiation therapy, late grade 2 toxicity did not differ significantly between the hypofractionation group (EQD2 3.3–84.2 Gy) and the conventional fractionation group (EQD2 74–86 Gy) (12% vs. 13%, *P* = 0.0878). In the NRG Oncology RTOG 0415 trial comparing two treatment schedules (70 Gy in 28 fractions vs. 73.8 Gy in 41 fractions), patients who received hypofractionated radiotherapy experienced more grade 2 and 3 late gastrointestinal adverse events than those who received conventionally fractionated radiotherapy (grade 2: 18.3% vs. 11.4%, *P* = 0.005; grade 3: 4.1% vs. 2.4%, *P* = 0.19, respectively) [29]. Compared to the hypofractionated radiotherapy group in RTOG 0415, who received the same treatment schedule as that used in the current study, patients in this study had a slightly lower rate of grade 2 toxicity (16.9% in this study vs. 22.4% in RTOG 0415) but a higher rate of grade 3 toxicity (9.5% in this study vs. 4.1% in RTOG 0415). This may be due to the relatively high proportion of anticoagulant users in this study.

Several previous studies have shown that patients taking anticoagulants have an increased risk of rectal bleeding following external beam radiotherapy for prostate cancer. According to a study by Takeda et al. that evaluated 232 patients who underwent radiotherapy for localized prostate cancer, the 50 patients who used anticoagulants had a 5-year incidence of grade 2 or 3 late rectal toxicity of 10.1%, compared to that of 4.3% in the 182 patients who did not take anticoagulants (*P* = 0.004) [14]. The total dose in that study ranged from 70–80 Gy, with 2.0 Gy per fraction. Choe et al. analyzed 568 patients who received definitive radiotherapy for prostate cancer, 79 of whom were taking an anticoagulant (warfarin or clopidogrel) [12]. That treatment schedule had a median total dose of 72.0 Gy, with 1.8 or 2.0 Gy per fraction. The anticoagulant group had a significantly higher risk of grade ≥ 3 bleeding than the non-anticoagulant group (4-year actuarial risk: 15.5% vs. 3.6%, risk ratio 2.51, 95% CI 1.72–3.66, *P* < 0.001). Schreiber et al. assessed the risk of late rectal bleeding in terms of the timing and type of anticoagulation used in 465 patients who received dose-escalated radiotherapy (minimum dose of 75.6 Gy, with 1.8 Gy per fraction). They found that patients on clopidogrel or warfarin during radiotherapy had a significantly increased risk of rectal bleeding, with a 4-year freedom from rectal bleeding rate of 75.2%, compared to that of 94.7% in those never on anticoagulation (HR 4.84, 95% CI 1.84–12.68, *P* = 0.001). However, anticoagulant use after radiotherapy (84 patients, 29.2% in total anticoagulant users) was not associated with a rectal bleeding risk of less than 10%. In this study, which evaluated patients who received hypofractionated radiotherapy, anticoagulant use was also significantly associated with a higher risk of grade ≥ 3 rectal bleeding, with an HR of 2.94. The relationship between the timing of anticoagulant use and rectal bleeding was not analysed because it was beyond the scope of this study. Further studies are needed to determine how the timing of anticoagulant use affects the risk of rectal bleeding. Another interesting point in our results is that the use of an anticoagulant was significantly associated only with grade ≥ 3 toxicity, not with grade 1–2 toxicity. It can thus be inferred that anticoagulants are associated with the severity rather than frequency of rectal bleeding; in other words, they serve to maintain or worsen rectal bleeding that has already begun, rather than acting as a trigger.

In addition to anticoagulant use, several previous studies have reported that advanced age, myocardial infarction, congestive heart failure, diabetes, hormone therapy, severe internal iliac artery calcification, and inflammatory bowel disease are significant patient-related risk factors for late rectal toxicity after radiotherapy for prostate cancer [11, 16, 17, 30–32]. In this study, unlike previous ones, advanced age and diabetes were not significantly related to late rectal bleeding. Instead, hypertension and liver cirrhosis were found to be prognostic factors for rectal bleeding. Hypertension probably increases vascular injury and intestinal ischemia following radiotherapy and impairs tissue repair [10]. Decreased platelet production, increased platelet destruction from hypersplenism, and decreased synthesis of clotting factors might explain the increased risk of rectal bleeding in cirrhotic patients [33, 34]. To our knowledge, this is the first study to find a significant correlation between cirrhosis and radiation-induced rectal toxicity. Further studies with more cirrhotic patients are needed to verify this hypothesis.

In this study, patients on anticoagulants and those with cirrhosis had a significantly higher risk of grade ≥ 3 rectal bleeding than those who did not, and EQD2 V77 Gy was the most predictive parameter for rectal bleeding in the anticoagulant group. For prostate cancer patients at high risk of rectal bleeding, hypofractionated dose-escalated radiotherapy should be used with caution. The use of rectal spacers, which increase the prostate–rectal interface, thereby reducing rectal toxicity, may benefit these patients [35]. In addition, it is necessary to minimize hotspots in patients on an anticoagulant.

Our conclusions are subject to the inevitable limitations of a retrospective analysis. First, the aims and treatment fields of radiotherapy were heterogeneous in the enrolled patients. Second, it was difficult to clarify which anticoagulants were more associated with rectal bleeding due to the variety of medications used. Third, it is unclear whether the rectal bleeding observed in patients on anticoagulants is caused by the anticoagulant itself or by other comorbidities that led to the taking of the anticoagulant. The strength of our study is that all included patients underwent hypofractionated radiotherapy using the intensity-modulation technique with daily image guidance, which has been widely used in recent years. Our results could be a good reference for other centers implementing similar treatment practices. In addition, grade ≥ 2 rectal bleeding was endoscopically confirmed to be related to radiation proctitis in all patients, improving the reliability of our results.

## Conclusions

In conclusion, patients taking anticoagulants and those with cirrhosis had a significantly higher risk of severe late rectal bleeding after hypofractionated radiotherapy for prostate cancer compared to other patients in the present study. Sufficient consultation about late complications of radiotherapy and other possible treatment options is necessary for patients with the aforementioned risk factors. The bleeding risk could be lowered by minimizing hotspots in patients on an anticoagulant.

## Supplementary Information

**Additional file 1: Table S1.** Statistical models in STATA and R software. **Table S2**. Variance ratio of covariate balance.

## Data Availability

The datasets used and/or analysed during the current study are available from the corresponding author on reasonable request.

## Abbreviations

EQD2: Equivalent dose at fractionation of 2 Gy
IRB: Institutional review board
PTV: Planning target volume
RTOG: Radiation therapy oncology group
gEUD: Generalized equivalent uniformed dose
IPTW: Inverse probability of treatment weighting
ROC: Receiver operator characteristic
AUC: Area under the curve
HR: Hazard ratio
CI: Confidence interval
3DCRT: 3-Dimensional conformal radiation therapy

## References

[1] Peeters ST, Heemsbergen WD, Koper PC, van Putten WL, Slot A, Dielwart MF (2006). Dose-response in radiotherapy for localized prostate cancer: results of the Dutch multicenter randomized phase III trial comparing 68 Gy of radiotherapy with 78 Gy. J Clin Oncol.

[2] Dearnaley DP, Sydes MR, Graham JD, Aird EG, Bottomley D, Cowan RA (2007). Escalated-dose versus standard-dose conformal radiotherapy in prostate cancer: first results from the MRC RT01 randomised controlled trial. Lancet Oncol.

[3] Catton CN, Lukka H, Gu CS, Martin JM, Supiot S, Chung PWM (2017). Randomized trial of a hypofractionated radiation regimen for the treatment of localized prostate cancer. J Clin Oncol.

[4] Dearnaley D, Syndikus I, Mossop H, Khoo V, Birtle A, Bloomfield D (2016). Conventional versus hypofractionated high-dose intensity-modulated radiotherapy for prostate cancer: 5-year outcomes of the randomised, non-inferiority, phase 3 CHHiP trial. Lancet Oncol.

[5] Incrocci L, Wortel RC, Alemayehu WG, Aluwini S, Schimmel E, Krol S (2016). Hypofractionated versus conventionally fractionated radiotherapy for patients with localised prostate cancer (HYPRO): final efficacy results from a randomised, multicentre, open-label, phase 3 trial. Lancet Oncol.

[6] Brenner DJ, Martinez AA, Edmundson GK, Mitchell C, Thames HD, Armour EP (2002). Direct evidence that prostate tumors show high sensitivity to fractionation (low alpha/beta ratio), similar to late-responding normal tissue. Int J Radiat Oncol Biol Phys.

[7] Fowler J, Chappell R, Ritter M (2001). Is alpha/beta for prostate tumors really low?. Int J Radiat Oncol Biol Phys.

[8] Sachpazidis I, Mavroidis P, Zamboglou C, Klein CM, Grosu AL, Baltas D (2020). Prostate cancer tumour control probability modelling for external beam radiotherapy based on multi-parametric MRI-GTV definition. Radiat Oncol.

[9] Kennedy GD, Heise CP (2007). Radiation colitis and proctitis. Clin Colon Rectal Surg.

[10] Grodsky MB, Sidani SM (2015). Radiation proctopathy. Clin Colon Rectal Surg.

[11] Hamstra DA, Stenmark MH, Ritter T, Litzenberg D, Jackson W, Johnson S (2013). Age and comorbid illness are associated with late rectal toxicity following dose-escalated radiation therapy for prostate cancer. Int J Radiat Oncol Biol Phys.

[12] Choe KS, Jani AB, Liauw SL (2010). External beam radiotherapy for prostate cancer patients on anticoagulation therapy: how significant is the bleeding toxicity?. Int J Radiat Oncol Biol Phys.

[13] Schreiber D, Chen SC, Rineer J, Worth M, Telivala T, Schwartz D (2014). Assessment of risk of late rectal bleeding for patients with prostate cancer started on anticoagulation before or after radiation treatment. Anticancer Res.

[14] Takeda K, Ogawa Y, Ariga H, Koto M, Sakayauchi T, Fujimoto K (2009). Clinical correlations between treatment with anticoagulants/antiaggregants and late rectal toxicity after radiotherapy for prostate cancer. Anticancer Res.

[15] Alashkham A, Paterson C, Hubbard S, Nabi G (2019). What is the impact of diabetes mellitus on radiation induced acute proctitis after radical radiotherapy for adenocarcinoma prostate? A prospective longitudinal study. Clin Transl Radiat Oncol.

[16] Maebayashi T, Ishibashi N, Aizawa T, Sakaguchi M, Sato H, Sato K (2017). Factors predicting late rectal disorders after radiation therapy for prostate cancer. Chin Med J (Engl).

[17] Ng BYH, Yu ELM, Lau TTS, Law KS, Cheng ACK (2019). Associations of clinical and dosimetric parameters with late rectal toxicities after radical intensity-modulated radiation therapy for prostate cancer: a single-centre retrospective study. Hong Kong Med J.

[18] Thames HD, Withers HR, Peters LJ, Fletcher GH (1982). Changes in early and late radiation responses with altered dose fractionation: implications for dose-survival relationships. Int J Radiat Oncol Biol Phys.

[19] Afkhami Ardekani M, Ghaffari H, Navaser M, Zoljalali Moghaddam SH, Refahi S (2021). Effectiveness of rectal displacement devices in managing prostate motion: a systematic review. Strahlenther Onkol.

[20] Smeenk RJ, Teh BS, Butler EB, van Lin EN, Kaanders JH (2010). Is there a role for endorectal balloons in prostate radiotherapy?. A systematic review Radiother Oncol.

[21] De Meerleer G, Berghen C, Briganti A, Vulsteke C, Murray J, Joniau S (2021). Elective nodal radiotherapy in prostate cancer. Lancet Oncol.

[22] Allen Li X, Alber M, Deasy JO, Jackson A, Ken Jee KW, Marks LB (2012). The use and QA of biologically related models for treatment planning: short report of the TG-166 of the therapy physics committee of the AAPM. Med Phys.

[23] Marzi S, Saracino B, Petrongari MG, Arcangeli S, Gomellini S, Arcangeli G (2009). Modeling of alpha/beta for late rectal toxicity from a randomized phase II study: conventional versus hypofractionated scheme for localized prostate cancer. J Exp Clin Cancer Res.

[24] Gulliford SL, Partridge M, Sydes MR, Andreyev J, Dearnaley DP (2010). A comparison of dose-volume constraints derived using peak and longitudinal definitions of late rectal toxicity. Radiother Oncol.

[25] Coia LR, Myerson RJ, Tepper JE (1995). Late effects of radiation therapy on the gastrointestinal tract. Int J Radiat Oncol Biol Phys.

[26] Viani GA, Stefano EJ, Afonso SL (2009). Higher-than-conventional radiation doses in localized prostate cancer treatment: a meta-analysis of randomized, controlled trials. Int J Radiat Oncol Biol Phys.

[27] Di Franco R, Borzillo V, Ravo V, Ametrano G, Cammarota F, Rossetti S (2017). Rectal/urinary toxicity after hypofractionated vs conventional radiotherapy in high risk prostate cancer: systematic review and meta analysis. Eur Rev Med Pharmacol Sci.

[28] Di Franco R, Borzillo V, Ravo V, Ametrano G, Falivene S, Cammarota F (2017). Rectal/urinary toxicity after hypofractionated vs conventional radiotherapy in low/intermediate risk localized prostate cancer: systematic review and meta analysis. Oncotarget.

[29] Lee WR, Dignam JJ, Amin MB, Bruner DW, Low D, Swanson GP (2016). Randomized phase III noninferiority study comparing two radiotherapy fractionation schedules in patients with low-risk prostate cancer. J Clin Oncol.

[30] Willett CG, Ooi CJ, Zietman AL, Menon V, Goldberg S, Sands BE (2000). Acute and late toxicity of patients with inflammatory bowel disease undergoing irradiation for abdominal and pelvic neoplasms. Int J Radiat Oncol Biol Phys.

[31] Kalakota K, Liauw SL (2013). Toxicity after external beam radiotherapy for prostate cancer: an analysis of late morbidity in men with diabetes mellitus. Urology.

[32] Giordano SH, Lee A, Kuo YF, Freeman J, Goodwin JS (2006). Late gastrointestinal toxicity after radiation for prostate cancer. Cancer.

[33] Flores B, Trivedi HD, Robson SC, Bonder A. Hemostasis, bleeding and thrombosis in liver disease. J Transl Sci 2017,3. 10.15761/JTS.1000182 PMC6136435 30221012

[34] Shi X, Xia H, Zhang W, Li G, Li A (2018). Radiotherapy for one rectal cancer patient with cirrhosis and moderate to severe thrombocytopenia: a case report. Onco Targets Ther.

[35] Payne HA, Pinkawa M, Peedell C, Bhattacharyya SK, Woodward E, Miller LE. SpaceOAR hydrogel spacer injection prior to stereotactic body radiation therapy for men with localized prostate cancer: A systematic review. 2021,100:e28111. 10.1097/MD.0000000000028111 PMC8663810 34889268

