# Supplementary material for: Patient-related risk factors for late rectal bleeding after hypofractionated radiotherapy for localized prostate cancer: a single-center retrospective study
Source: Radiat Oncol. 2022 Feb 9;17:30. doi: 10.1186/s13014-022-01998-4 (PMC8827292; doi:10.1186/s13014-022-01998-4)
Supplement: Supplementary file 1 — Additional file 1: Table S1. Statistical models in STATA and R software. Table S2. Variance ratio of covariate balance. [file 13014_2022_1998_MOESM1_ESM.docx]

Table S1. Statistical models in STATA and R software

| Statistical package | Statistical model | Application |
| --- | --- | --- |
| STATA | Logistic regression analysis  Pearson's chi-squared testing  Inverse probability of treatment weighting and propensity scoring  Propensity-weighted log-rank test | Table 2 Table 3  Table 3  Fig. 1 |
| R | Receiver operating characteristic curve analysis | Table 4 |

| Table S2. Variance ratio of covariate balance | | |
| --- | --- | --- |
| Variables | Variance ratio | |
|  | Raw | Weighted |
| Age (years) (< 74 vs. ≥ 74) | 0.948 | 1.005 |
| Risk category (very low–low vs. intermediate vs. high–very high) | 1.305 | 1.020 |
| Gleason score (2–6 vs. 7 vs. 8–10) | 1.093 | 1.018 |
| Initial PSA concentration (μg/L) (<10 vs. 10–20 vs. ≥20) | 0.988 | 1.089 |
| T stage (T1–2 vs. ≥ T3) | 1.151 | 1.020 |
| Diabetes (yes vs. no) | 1.985 | 0.970 |
| Hypertension (yes vs. no) | 0.917 | 1.000 |
| Cirrhosis (yes vs. no) | 0.638 | 1.201 |
| Whole-pelvic radiotherapy (yes vs. no) | 0.868 | 0.933 |
| Androgen-deprivation therapy (yes vs. no) | 1.251 | 1.000 |
| Radiation dose (Gy) (67.2 vs. 70) | 1.062 | 0.986 |
| IMRT technique (static vs. arc) | 1.098 | 1.007 |

IMRT, intensity-modulated radiation therapy; PSA, prostate-specific antigen.
